# CUB Domain Containing Protein 1 (CDCP1) modulates adhesion and motility in colon cancer cells

**DOI:** 10.1186/1471-2407-14-754

**Published:** 2014-10-09

**Authors:** David J Orchard-Webb, Thong Chuan Lee, Graham P Cook, G Eric Blair

**Affiliations:** School of Molecular and Cellular Biology, Garstang Building, University of Leeds, Leeds, LS2 9JT UK; Leeds Institute of Cancer and Pathology, Wellcome Brenner Building, St. James’s University Hospital, University of Leeds, Leeds, LS9 7TF UK; School of Molecular and Cellular Biology, Faculty of Biological Sciences, University of Leeds, Garstang Building, Room 8.53f, Leeds, LS2 9JT UK

**Keywords:** CDCP1, CD9, Motility, Adhesion

## Abstract

**Background:**

Deregulated expression of the transmembrane glycoprotein CDCP1 (CUB domain-containing protein-1) has been detected in several cancers including colon, lung, gastric, breast, and pancreatic carcinomas. CDCP1 has been proposed to either positively or negatively regulate tumour metastasis. In this study we assessed the role of CDCP1 in properties of cells that are directly relevant to metastasis, namely adhesion and motility. In addition, association between CDCP1 and the tetraspanin protein CD9 was investigated.

**Methods:**

CDCP1 and CD9 protein expression was measured in a series of colon cancer cell lines by flow cytometry and Western blotting. Adhesion of Colo320 and SW480 cells was determined using a Matrigel adhesion assay. The chemotactic motility of SW480 cells in which CDCP1 expression had been reduced by RNA interference was analysed using the xCELLigence system Real-Time Cell Analyzer Dual Plates combined with 8 μm pore filters. Detergent-resistant membrane fractions were generated following density gradient centrifugation and the CDCP1 and CD9 protein composition of these fractions was determined by Western blotting. The potential association of the CDCP1 and CD9 proteins was assessed by co-immunoprecipitation.

**Results:**

Engineered CDCP1 expression in Colo320 cells resulted in a reduction in cell adhesion to Matrigel. Treatment of SW480 cells with CDCP1 siRNA reduced serum-induced chemotaxis. CDCP1 and CD9 cell-surface protein and mRNA levels showed a positive correlation in colon cancer cell lines and the proteins formed a low-level, but detectable complex as judged by co-sedimentation of detergent lysates of HT-29 cells in sucrose gradients as well as by co-immunoprecipitation in SW480 cell lysates.

**Conclusions:**

A number of recent studies have assigned a potentially important role for the cell-surface protein CDCP1 in invasion and metastasis of a several types of human cancer cells. In this study, CDCP1 was shown to modulate cell-substratum adhesion and motility in colon cancer cell lines, with some variation depending on the colon cancer cell type. CDCP1 and CD9 were co-expressed at the mRNA and protein level and we obtained evidence for the presence of a molecular complex of these proteins in SW480 colon cancer cells.

**Electronic supplementary material:**

The online version of this article (doi:10.1186/1471-2407-14-754) contains supplementary material, which is available to authorized users.

## Background

CUB domain-containing protein-1 (CDCP1, also termed CD318, SIMA135 and Trask) is an 836 amino acid type I integral membrane glycoprotein that may play a role in cancer metastasis [1]. Increased CDCP1 expression has been found in prostate and squamous cell carcinoma cell lines with high metastatic potential [1, 2]. Deregulated CDCP1 expression has been detected in colon, lung, gastric, breast and pancreatic carcinomas, compared to normal tissues [1, 3–7]. Elevated CDCP1 expression in tumour biopsies has been associated with reduced patient survival in pancreatic, lung and renal cell carcinoma [3, 8, 9]. CDCP1 has also been shown to affect cell migration and adhesion *in vitro* as well increasing metastasis of cancer cell lines in certain *in vivo* model systems [1, 6, 9–11]. However there is also evidence from mouse model systems that CDCP1 may repress metastasis using xenografts of human breast, pancreatic and fibroblastic cell lines in which overexpression of CDCP1 has been engineered [12]. It is possible that the apparent differences in the effect of CDCP1 on metastasis are due to the model system used.

CDCP1 has been shown to play a role in cell motility and adhesion of certain cancer cell lines. It directly interacts with proteins involved in both cell-cell and cell-ECM adhesion. CDCP1 has been shown to co-immunoprecipitate with the adherens junction proteins N- and P-cadherin and the focal adhesion proteins syndecans 1 and 4 [13]. Consistent with this, a number of studies have shown that CDCP1 modulates adhesion of cancer cell lines to an extracellular matrix (ECM) [6, 10]. Treatment of the colon cancer cell line DLD-1 with an anti-CDCP1 antibody resulted in the stimulation of cell migration through filters [14]. Reduction of CDCP1 by RNA interference in the pancreatic cancer cell line BxPc3 and the gastric cancer cell lines 44As3 and 58As9 decreased cell migration and invasion through Matrigel of [3, 6]. In contrast, engineered over-expression of CDCP1 in the gastric cancer cell lines HSC59 and HSC60 increased cell migration [6].

Tetraspanin proteins are approximately 25 kDa integral membrane proteins that contain four membrane-spanning domains, with a distinctive large and small extracellular loop that distinguishes them from other four span membrane proteins [15]. There are 33 human tetraspanin genes and their proteins are thought to regulate the function of binding partner proteins and coordinate their localisation within the plasma membrane [16]. The totality of tetraspanin interactions has been termed the "tetraspanin web" [17–19]. Proteomic and immunofluorescence-based approaches have suggested that CDCP1 and the tetraspanin CD9 could be located within the tetraspanin web [20, 21]. However this proposal has not been confirmed by co-immunoprecipitation or co-localisation in membrane fractions.

The expression of CDCP1 and CD9 proteins has not been extensively characterised in colon cancer cell lines. The purpose of this study was to perform a molecular characterisation of CDCP1 and CD9 protein expression in a panel of colon cancer cell lines and, given the proposed role of CDCP1 in metastasis, to assess the effect of CDCP1 expression on properties of these cancer cells that are directly relevant to metastasis, namely adhesion and motility.

## Methods

### Materials

Mouse monoclonal anti-CDCP1 clone CUB1 was from MBL International (Woburn, MA, U.S.A.). Goat polyclonal anti-CDCP1 (Ab1377) was from Abcam (Cambridge, U.K.). Mouse monoclonal CD9 clone ALB6 (sc-59140) was from Santa Cruz (Insight Biotechnology Ltd, Wembley, U.K.) Mouse monoclonal anti-CD9 antibody clone 602–29 was generated by Andrews et al. [22] and was a kind gift from Drs Peter Monk and Lynda Partridge (University of Sheffield). Monoclonal mouse anti- Flotillin-1 clone 18 (610820) was from BD Transduction Laboratories (Oxford, U.K.). Monoclonal mouse anti-Transferrin Receptor clone H68.4 (13–6800) and Alexa 488-conjugated goat anti-mouse IgG antibody (A-11029) were from Invitrogen (Paisley, U.K.). Rabbit anti-goat IgG conjugated to HRP (A5420) and sheep anti-mouse IgG conjugated to HRP (A6782) were from Sigma (St. Louis, MO, U.S.A.). Monoclonal mouse anti-GAPDH clone 6C5 was from Calbiochem (Merck Chemicals Ltd., Nottingham, U.K.). Mouse IgG1 isotype control (401402) was from Biolegend (Cambridge Bioscience, Cambridge, U.K.). Protein G beads (20398) and HALT protease inhibitor cocktail (78415) were from Pierce (Perbio Science UK, Ltd, Cramlington, U.K.). Triton X-100 (T9284), Brij58 (P5884), Brij97 (P6136), were from Sigma (Dorset, U.K.). Ultraclear ½ × 2 inch centrifuge tubes were from Beckman (High Wycombe, U.K.). Basement Membrane Matrix (BD Matrigel™ - 356234) was purchased from BD Biosciences Discovery Labware (Oxford, U.K.). Kwill Filling tubes (UN888) were from Universal Hospital Supplies (Enfield London, U.K.). Lipofectamine 2000 was purchased from Invitrogen (Paisley, U.K.). G418 was from Melford (Chelsworth, U.K.). Cell Invasion and Motility (CIM) plates (16-well, 8 μm pore filter plates) and Real-Time Cell Analyzer dual plates (RTCA DP) were from Roche (Burgess Hill, U.K.).

### Cell lines and culture

Colo320 (isolated from an undifferentiated Dukes’ C colorectal adenocarcinoma), Colo741 (isolated from a pelvic wall metastasis of a colorectal tumour), SW480 (isolated from a Dukes’ B colorectal adenocarcinoma), HCT116 (isolated from a primary colonic tumour), HT-29 (isolated from a primary colonic tumour), HaCaT (a spontaneously transformed keratinocyte cell line isolated from histologically normal human skin) and A549 (a human alveolar basal epithelial cell line isolated from a bronchial adenocarcinoma) cells were maintained in Dulbecco’s Modified Eagle’s Medium (DMEM) supplemented with 10% (v/v) foetal bovine serum (FBS) and 2 mM L-Glutamine (10% FBS DMEM) at 37°C in a humidified atmosphere containing 7.5% CO_2_. Cell lines were purchased from ECACC or ATCC with the exception of HaCaT cells which were kindly donated by Dr Miriam Wittmann (Leeds Institute of Rheumatic and Musculoskeletal Medicine, University of Leeds, Leeds, UK).

### Generation of stable CDCP1-expressing Colo320 cell lines

A CDCP1-FLAG-pcDNA3.1+ expression plasmid was kindly donated by Stephen P. Soltoff [23]. A six well plate was seeded in antibiotic-free 10% FBS DMEM with 5×10^5^ Colo320 cells. Cells were allowed to grow overnight. Two micrograms of CDCP1-FLAG construct or pcDNA3 was added to 250 μl OptiMEM. Ten microlitres of Lipofectamine 2000 was diluted into 250 μl of OptiMEM and incubated for five minutes. The diluted plasmid was added to the diluted Lipofectamine and incubated at room temperature for 20 minutes. The growth medium in Colo320 cells was replaced with serum-free DMEM. The 2 μg plasmid/Lipofectamine/OptiMEM mixture was added to wells in a volume of 500 μl. After six hours, the medium was changed to 10% FBS DMEM. The cells were allowed to grow for 48 hours before 10% FBS DMEM containing 1.5 mg/ml G418 (final concentration) was added to the wells. The Colo320 cells were selected for five days before counting the cells and plating in a 1:1 mixture of fresh 10% FBS DMEM and cell-free conditioned medium (which comprised 10% DMEM previously used to grow Colo320 cells) at an average of 1 cell per well in 96 well plates. The cells were maintained in G418 and grown for approximately four weeks. Once colonies were of sufficient size they were transferred to 6-well plates, followed by T-25 flasks maintained in the G418 medium. Twelve such G418-resistant clones were obtained. The clones were screened for cell-surface CDCP1 expression by flow cytometry.

### RNA interference

Duplex siRNA was synthesised by Eurogentec (Hampshire, U.K.). The complementary (upper) sequences in the two RNA duplexes were: Control (5′-ACG-UGA-CAC-GUU-CGG-AGA-A_TT_-3′) and CDCP1 oligo 2 (5′- GUC-CUG-AGA-AUC-ACU-UUG-U_TT_-3′). For transfection, cells were plated at 2.2 × 10^5^ cells per well in 6-well plates. Cells were grown for 16 hours until they were approx. 30% confluent. The siRNA was diluted into 250 μl OptiMEM such that the final siRNA concentration was 8nM. Lipofectamine 2000 (2.5 μl) was diluted into 250 μl OptiMEM, mixed gently and incubated at room temperature for 5 minutes. The diluted siRNA was added to the Lipofectamine 2000 dilution and incubated for 20 min at room temperature. Cells were washed twice in 1 ml of PBS. Serum-free DMEM (2 ml) containing 2 mM L-glutamine was added to each well. The 500 μl aliquots of siRNA-Lipofectamine 2000 complexes were added to each well. The medium was mixed by rocking and incubated for 6 h at 37°C. The medium was replaced with 10% FBS DMEM and incubated at 37°C for 48 hours before further analysis.

### Detection of cell-surface molecules by flow cytometry (FACS)

Cells were plated in triplicate at 6 × 10^5^ per well in 6 well plates. They were grown for 24 h until confluent. Cells were detached with Trypsin, quenched with 10% FBS-DMEM and pelleted by centrifugation in 1.5 ml microfuge tubes at 350 g, 4°C for 3 minutes. Cells were resuspended at 5 × 10^5^ per 50 μl in 10% NGS (normal goat serum) FACS buffer (1% (w/v) BSA, 1 mM EDTA, 25 mM HEPES-KOH pH 7.4 in PBS). Cells (5 × 10^5^) were incubated in 10% NGS FACS buffer for 15 min on ice, centrifuged and the pellets resuspended in 10% NGS FACS buffer containing either 10 μg/ml monoclonal mouse anti-CDCP1 clone CUB1 or 40 μg/ml monoclonal mouse anti-CD9 clone ALB6 or corresponding isotype-matched control antibodies. Cells were incubated for 30 min on ice, centrifuged and washed in 50 μl FACS buffer. Cells were resuspended in 50 μl 10% NGS FACS buffer containing 2 μg/ml Alexa 488-conjugated goat anti-mouse IgG antibody (Invitrogen A-11029) and incubated for 30 min on ice. Cells were centrifuged and pellets resuspended in 500 μl PBS. Cells were analysed on a Becton Dickinson FACScalibur flow cytometer using CELLQuest software.

### Western blotting

Cells were washed twice in PBS on ice and scraped into 200 μl RIPA lysis buffer (50 mM Tris–HCl pH 8.0, 150 mM NaCl, 0.1% (w/v) SDS, 25 mM EDTA, 2 mM DTT, 1% Imbentin-N/52 (NP-40 Substitute) containing fresh 1× HALT protease inhibitor cocktail (Pierce 78415, Perbio Science UK Ltd, Cramlington, UK). The lysates were homogenised by passage through a 25G syringe five times, centrifuged at 18 000 g at 4°C and the supernatants stored at -20°C. Protein concentration was determined by the BCA assay (Perbio Science UK Ltd, Cramlington, UK). Samples were diluted 1:1 in 2× Laemmli buffer (125 mM Tris-HC1 pH 6.8, 4% (w/v) SDS, 20% (v/v) glycerol, 0.01% (v/v) bromophenol blue, +/- 5% (v/v) β-mercaptoethanol), boiled for 5 min and placed immediately on ice. Samples were loaded onto 5% stacking, 10% or 12% separating polyacrylamide gels, applying 10-15 μl per well. Electrophoresis was performed in running buffer (25 mM Tris-base, 192 mM glycine, 0.1% (w/v) SDS) at 100 V for 20 min followed by 145 V until completion. Protein was transferred from the gel to methanol-activated PVDF membranes (Millipore, UK) by wet or semi-dry transfer in transfer buffer (25 mM Tris-base, 192 mM glycine, 10% (v/v) methanol). Wet transfer was performed at 100 V for 1 h in a cold room. Semi-dry transfer was performed at 12 V for 1.5 h at room temperature. The PVDF membranes were washed three times for 5 min in TBST (20 mM Tris–HCl pH 7.6, 136 mM NaCl, 0.1% (v/v) Tween-20). Membranes were blocked with 5% Marvel milk powder in TBST for one hour at room temperature. The primary antibody was added in 1 - 10 ml of 5% Marvel milk powder in TBST and incubated overnight in the cold room. The PVDF membranes were washed three times for 5 min in TBST followed by the addition of HRP-conjugated secondary antibody in 5% milk TBST with incubation for one hour at room temperature. The membranes were washed three times for 5 min each in TBST and finally for 20 min in TBST. Equal volumes of Amersham ECL Plus™ detection solutions A and B were mixed and applied to the membranes. Images were captured using a chemiluminescence imager (Fuji LAS-3000, Japan) and analysed using AIDA software (Raytek Scientific Ltd, Sheffield, UK).

### Immunoprecipitation

SW480 cells were plated at 5 × 10^5^ cells/ well in 6-well plates and allowed to grow for approximately two complete days. The cells were washed twice in wash buffer (10 mM Tris–HCl pH 7.4, 150 mM NaCl, 1 mM CaCl_2_, 1 mM MgCl_2_) and scraped into lysis buffer (wash buffer plus 1% Brij97 (w/v) and fresh 1× HALT protease inhibitor cocktail. Lysates were centrifuged at 10,000 g for 10 minutes at 4°C. The protein concentration of the supernatants was determined by the Lowry assay (Biorad Dc assay cat: 500, Biorad, UK). Aliquots of cell lysates containing 500 μg protein were transferred to new tubes and the volume adjusted to 400 μl with lysis buffer. The lysates were pre-cleared with 10 μl protein G beads (Pierce, UK), previously washed and resuspended in lysis buffer, with rotation for two hours at 4°C. The beads were pelleted by centrifugation at 14000 g for 10 minutes at 4°C and the supernatant was transferred to new tubes. Mouse anti-CD9 (clone ALB6) (5 μg), or isotype-matched mouse control IgG (5 μg), and 50 μl of protein G beads were added to the lysates and rotated overnight at 4°C. The beads were pelleted at 2000 g for one minute at 4°C and washed four times in wash buffer supplemented with 1× HALT and boiled for 5 minutes in 40 μl 2× Laemmli loading buffer (without reducing agent). The lysate was divided into aliquots and, dependent on the antibody used for protein detection in the Western blot analysis, β-mercaptoethanol was added to the samples and boiled for a further 5 min before loading on SDS-PAGE gels. For detection of CDCP1, total protein was transferred to PVDF by wet transfer, semi-dry transfer was used for detection of CD9 and EpCAM. CDCP1 protein was detected with 4 μg goat anti-CDCP1 (Abcam ab1377) incubated at 4°C overnight. CD9 was detected with 4 μg mouse anti-CD9 (clone 602.29). EpCAM was detected with 2 μl rabbit anti-EpCAM (Abcam ab32392; antibody concentration not specified).

### Detergent-resistant membrane fractionation

HT-29 cells were grown in T-175 flasks until confluent. All steps were performed at 4°C. Each flask was washed once with 20 ml ice cold MBS (25 mM Mes-NaOH pH 6.5, 150 mM NaCl). Cells were scraped into 2 ml of lysis buffer (MBS containing either 0.5% (w/v) Triton X-100, Brij58 or Brij97 and HALT protease inhibitor cocktail). The lysates were homogenised by passing through a 25 g needle five times. Debris was pelleted by centrifugation at 500 g for 5 minutes at 4°C. Equal volumes of cleared lysate were combined with 80% (w/v) sucrose-MBS. One ml of the (40%) sucrose lysate was placed in the bottom of Beckman Ultraclear ½ × 2 inch centrifuge tubes. A sucrose step gradient was generated by layering 3 ml of 30% sucrose MBS and 1 ml of 5% sucrose MBS sequentially on top using Kwill Filling tubes. The tubes were transferred to an SW-55 rotor and centrifuged at 140,000 g for 18 h at 4°C in a Beckman Coulter Optima L-80 Ultracentrifuge. Eleven fractions (0.5 ml) were removed from the top of the tube using a cut P1000 pipette tip i.e. fraction 1 was the top and fraction 11was the bottom fraction of the gradient. The fractions were frozen at -20°C prior to Western blot analysis.

### Cell adhesion assay

An aliquot of basement membrane matrix (BD Matrigel™) was thawed overnight at 4°C. The Matrigel was diluted to 10 μg/ml in serum-free ice-cold DMEM using pipette tips that had been cooled to -20°C. The Matrigel solution (500 μl/ well) was added to 24-well plates and incubated for one hour at room temperature. The medium was aspirated and cells were immediately added. Cells were plated at 2 × 10^5^ cells per well of 24 well plates in 500 μl 10% FBS DMEM. In order to generate an adhesion index (from protein quantification), 2 × 10^5^ cells (termed reference cells) in 10% FBS DMEM were retained in 1.5 ml microfuge tubes and incubated alongside the test plates. The cells were incubated for one hour at 37°C. Plates were washed once with 200 μl PBS. The remaining cells were lysed in 50 μl of RIPA buffer (50 mM Tris–HCl pH 8.0, 150 mM NaCl, 0.1% (w/v) SDS, 25 mM EDTA, 2 mM DTT, 1% (v/v) Imbentin-N/52 (NP-40 Substitute)) by shaking for one hour at 4°C. The reference cell aliquots in microfuge tubes were centrifuged at 500 g at 4°C for 3 minutes and lysed in 50 μl RIPA by resuspension and shaking at 4°C for one hour. The lysate protein content was quantified by the BCA assay in 96 well plates. An adhesion index was generated by calculating the fraction of protein (relative to the reference cell protein content) remaining in the wells after washing in PBS.

### Cell motility assay

Cell motility was analysed using the xCELLigence system Real-Time Cell Analyzer Dual Plate (RTCA DP) (Roche) [24]. The RTCA DP is a device that monitors electrical impedance on the surface of specially designed tissue culture plates. The greater the surface area of the plate covered by cells the larger the electrical impedance. Therefore cell number can be inferred from the electrical impedance, with the caveat that cell morphology and adhesive strength also influences the electrical impedance. Roche Cell Invasion and Motility (CIM)-plate 16 s (16-well, 8 μm pore filter plates) tissue culture plates were used to assess cell motility. These plates are similar to standard trans-well filter plates; however they contain microelectrodes on the lower surface of the filter from which electrical impedance is measured. This impedance should increase as cells migrate through the filter to the lower surface, reaching a maximum when the lower filter surface is fully covered. The baseline impedance is set by taking a reading with no cells and only medium in the plate. The RTCA DP was maintained at 37°C inside a CO_2_ incubator. Prior to motility analysis, the medium of SW480 cells transfected with control or CDCP1 siRNA was replaced with serum-free DMEM and incubated for 30 minutes at 37°C. CIM-plate 16 s were prepared containing 150 μl serum-free DMEM or 10% FBS DMEM in the lower well. The upper wells were attached and 100 μl of serum-free DMEM was added per well. The CIM plates were incubated at 37°C for approximately one hour. Cells for analysis were removed from flasks by treatment with Versene at 37°C for 20 minutes. Serum-free DMEM (1 ml) was added to the suspended cells which were counted. A baseline impedance reading was taken using the cell-free CIM plates. Cells were added to the upper wells of the CIM plates in 48 μl serum-free medium to give 40,000 cells per well. The CIM plates were placed in the xCELLigence system Real-Time Cell Analyzer (RTCA) DP and allowed to incubate for 72 h. A reading of the impedance on the underside of the 8 μm pore membrane was taken every 10 minutes and reported as a dimensionless Cell Index (CI) which is derived from the relative change in electrical impedance set against the baseline reading (baseline CI =0).

## Results

### CDCP1 protein expression in colon cancer cell lines and its effects on adhesion and motility

**Figure 1:**
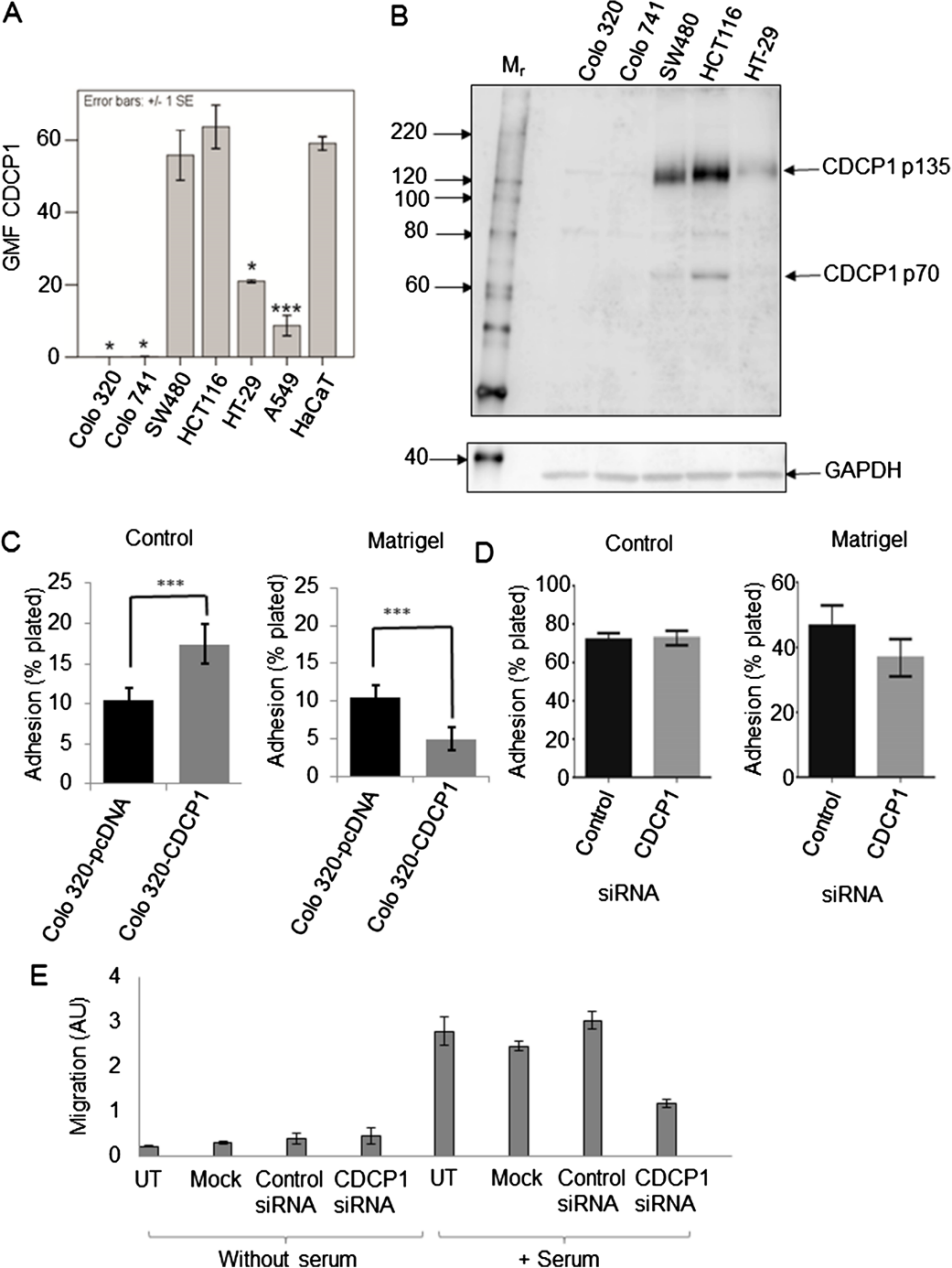
**CDCP1 protein expression in colon cancer cell lines and modulation of adhesion and motility. A)** Cell-surface expression of CDCP1 was measured by flow cytometry using monoclonal mouse anti-CDCP1 clone CUB1. Bound primary antibody was detected with Alexa488-conjugated goat anti-mouse IgG. Standard error bars are shown. n ≥3, except HaCaT where n =2. Cell lines are indicated that showed significantly different CDCP1 cell-surface expression from HaCaT as determined by independent sample 2-tailed t-tests not assuming equal variance. GMF CDCP1, geometric mean fluorescence of bound anti-CDCP1. *p ≤0.05; *** = p ≤0.001. **B)** Western blot for CDCP1. Total cell protein (40 μg) was separated by SDS-PAGE, transferred to PVDF and CDCP1 detected using 1 μg/ml goat polyclonal antibody anti-CDCP1 antibody (ab1377). The membrane was re-probed for GAPDH to assess protein loading. The image is representative of three blots from independent lysates. **C)** Binding of stable CDCP1-expressing Colo320 (Colo320-CDCP1) and control Colo320 (Colo320-pcDNA3) cells to control or 10 μg/ml Matrigel coated tissue culture plates. The Colo320 cell-substratum assays were repeated four times. *** = p ≤0.001. **D)** Adhesion of SW480 cells to control or Matrigel-coated tissue culture plates following reduction of CDCP1 by RNA interference. The experiment was repeated three times. Control, cells transfected with control siRNA; CDCP1, cells transfected with CDCP1 siRNA oligo 2 (see Additional file 1: Figure S2). **E)** Motility of SW480 cells through an 8 μm pore membrane towards serum following reduction of CDCP1 by RNA interference. This was measured in arbitrary units (AU) at 60 h after siRNA transfection using the xCELLigence system Real-Time Cell Analyzer Dual Plate (RTCA DP, Roche). UT, untransfected cells; mock, cells treated with Lipofectamine 2000 only; Control siRNA, cells transfected with CDCP1 siRNA oligo 2. See Additional file 1: Figure S3 for a more extensive kinetic analysis.

Cell-surface expression of the CDCP1 protein was examined by flow cytometry (Figure 1A). CDCP1 mRNA was first identified as up-regulated, compared to normal lung tissue, in the lung cancer cell line A549 and so this cell line was included for comparison [5]. The expression of cell-surface CDCP1 was compared to the non-tumorigenic immortalised keratinocyte cell line HaCaT by independent sample 2-tailed t-tests not assuming equal variance (Figure 1A). None of the colon cancer cell lines had significantly higher CDCP1 expression than HaCaT. Significantly lower expression was found in A549 cells and the colon cancer cell lines HT-29, Colo741 and Colo320. In Colo320 and Colo741 cells which have a non-epithelial morphology (Colo320 – rounded exocrine and Colo741 – fibroblastic), CDCP1 protein was not detected at the cell surface, consistent with the proposal that CDCP1 is primarily expressed in epithelial cells [12].

Total cellular CDCP1 protein expression was examined in colon cancer cell lines by Western blotting (Figure 1B). This analysis recapitulated the flow cytometry data. There were two endogenous CDCP1 protein species of approximately 135 kDa and 70 kDa termed p135 and p70 respectively, as previously reported [25, 26].

Having established the CDCP1 expression status of the colon cancer cell lines, CDCP1 expression was engineered in Colo320 cells by stable transfection of a CDCP1-FLAG-pcDNA3.1 plasmid (Additional file 1: Figure S1). Twelve clones of G418-resistant cells were screened by flow cytometry and, where relevant, Western blotting for CDCP1 expression. Clone 12 was used for all further experiments on CDCP1-expressing Colo320 cells. A G418-resistant clone generated by transfection with the cloning vector, pcDNA3, was used as a negative control Colo320 cell line. Ectopic expression of CDCP1 in Colo320 changed the adherence of the transfected cells. Interestingly, CDCP1 expression enhanced adhesion to conventional tissue culture plates but adherence to Matrigel was significantly decreased in comparison to control Colo320 cells, as previously observed for CDCP1-expressing HeLa cells [10] (Figure 1C).

A converse experiment was performed in which CDCP1 protein was reduced in SW480 cells by RNA interference (Additional file 1: Figure S2), giving an approx. 85% and 65% reduction in cell-surface and total cellular populations of CDCP1 respectively (data not shown). However, siRNA-mediated reduction of CDCP1 expression did not significantly alter binding to Matrigel (Figure 1D). Thus, although over expression of CDCP1 in Colo320 decreased the binding to Matrigel, a reduction in endogenous CDCP1 expresion did not alter the binding to Matrigel in the SW480 background, suggesting that the effect of CDCP1 on ECM adhesion may be colon cancer cell line-specific.

Cell motility is important for cancer cell metastasis and CDCP1 has been implicated in the motility of other cancer cell lines. The effect of reducing CDCP1 protein in SW480 cells by RNA interference on chemotactic migration towards serum was examined using the xCELLigence Real-Time Cell Analyzer Dual Plate (RTCA DP) system combined with 8 μm pore filter CIM plates (Roche) [24]. There was a marked decrease in SW480 cell motility through 8 μm pore filters towards serum when the cells were treated with CDCP1 siRNA (Figure 1E and Additional file 1: Figure S3). CDCP1 reduction by RNA interference in SW480 cells had no effect on initial cell adhesion to control cell culture plates (Figure 1D), therefore the effect on cell motility is unlikely to be due to differences in the rate of initial cell attachment to the filter.

### CD9 expression in colon cancer cell lines

**Figure 2:**
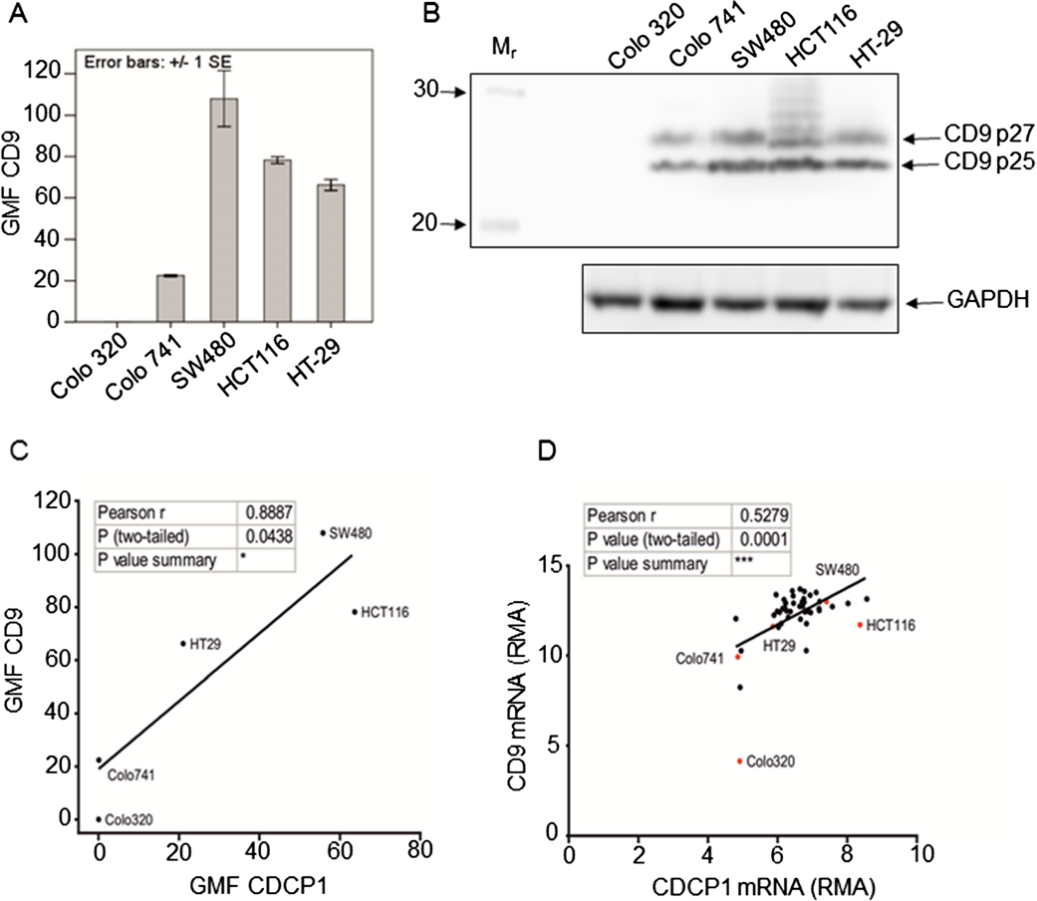
**CDCP1 and CD9 expression in colon cancer cell lines. A)** Cell-surface CD9 expression was measured by flow cytometry using monoclonal mouse anti-CD9 clone ALB6. The primary antibody was detected with Alexa488-conjugated goat anti-mouse IgG. Ten thousand cells were gated per sample. Standard error bars are shown. GMF, geometric mean fluorescence. **B)** Western blot analysis of CD9 expression. Total cell protein (40 μg) in RIPA buffer was separated by SDS-PAGE, transferred to PVDF and CD9 detected using 2 μg/ml mouse monoclonal antibody clone 602–29. Subsequently the PVDF membrane was stripped and re-probed for GAPDH to assess loading. The image is representative of three blots. Arrows indicate the locations of the CD9 species termed p27 and p25. **C)** An XY plot of the average CDCP1 and CD9 cell surface expression per cell line. A line of best fit was generated by linear regression. There is a significant positive correlation by bivariate Pearson correlation analysis. *p =0.044. GMF, geometric mean fluorescence. **D)** CDCP1 and CD9 mRNA expression determined by microarray (Robust multi-array average (RMA)) from 47 colon cancer cell lines was downloaded from the Cancer Cell Line Encyclopedia [28]. An XY plot was generated. The cell lines that were experimentally tested for cell-surface CDCP1 and CD9 protein expression are highlighted in red. A line of best fit was generated by linear regression. There is a highly significant positive correlation as reported by a bivariate Pearson correlation analysis. ***p < 0.001.

Cell-surface CD9 was detected in all colon cancer cell lines with the exception of Colo320 (Figure 2A). CD9 protein was present as two species migrating at 25 kDa (p25) and 27 kDa (p27) (Figure 2B). This is due to N-glycosylation of p27 that is absent in p25 [27]. A significant positive correlation (p = 0.044) was found between the CDCP1 and CD9 cell-surface protein expression patterns by Pearson correlation analysis (Figure 2C). Consistent with this, analysis of data obtained from the Cancer Cell Line Encyclopedia [28] ( http://www.broadinstitute.org/ccle/home) of 47 colon cancer cell lines for mRNA expression reported as a robust multi-array average (RMA) of CDCP1 and CD9 showed a highly significant positive correlation (p = 0.0001) between CDCP1 and CD9 mRNA expression by Pearson correlation analysis (Figure 2D). For ease of comparison with the cell-surface protein data (Figure 2C), those cell lines in which CDCP1 and CD9 protein expression were experimentally examined are highlighted in red and labelled (Figure 2D). A complete listing of the cell lines and their CDCP1 and CD9 RMA values reported in Figure 2D is shown in Additional file 2: Table S1. Of importance for CDCP1 and CD9 studies, the Colo320 cell line did not express either protein at the cell-surface and had correspondingly low mRNA expression (Figures 2C and D) [4].

### Localisation of CDCP1 and CD9 in detergent-resistant membrane (DRM) fractions in HT-29 cells

**Figure 3:**
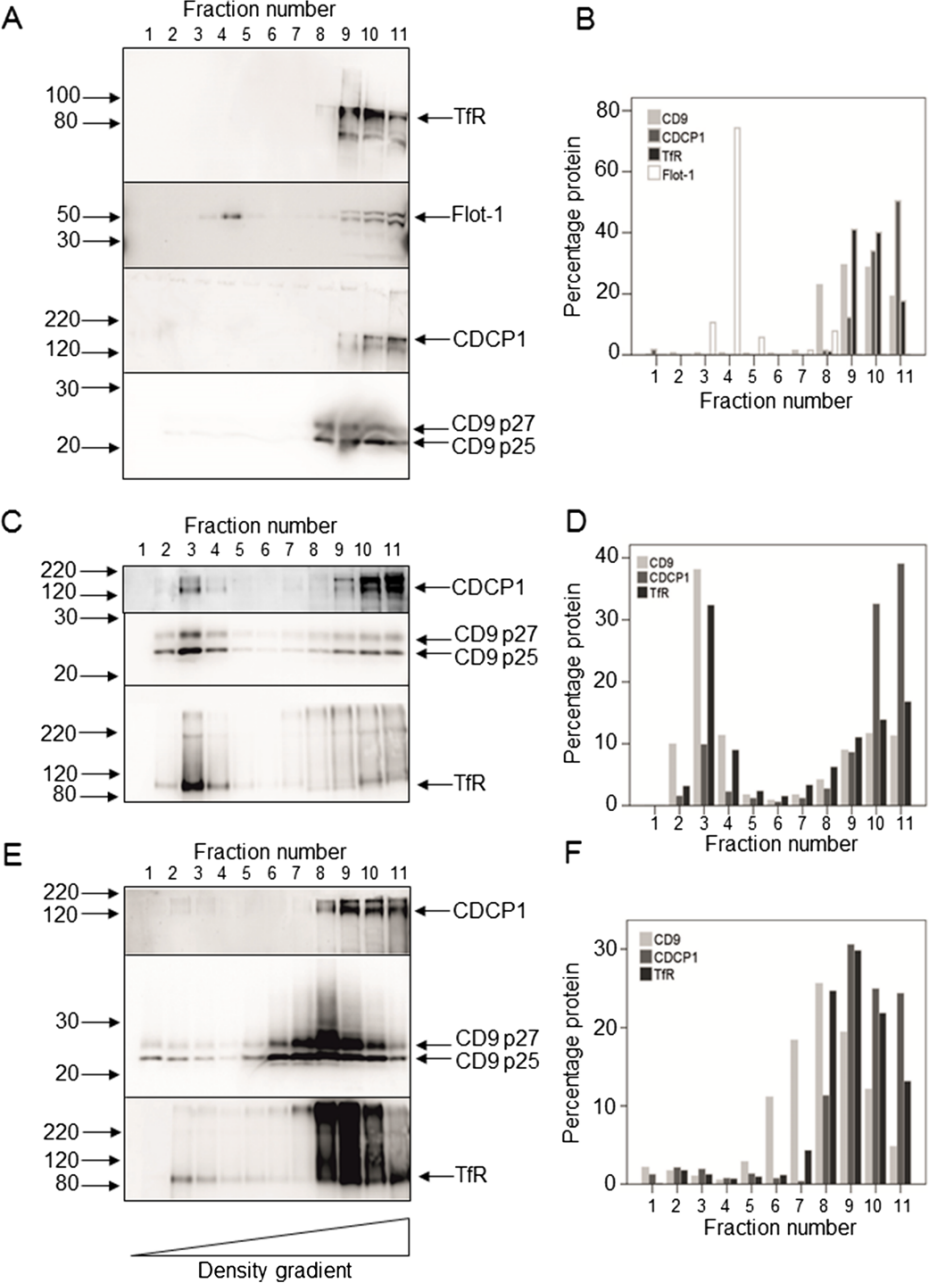
**Analysis of CDCP1 and CD9 in detergent-resistant membrane (DRM) fractions of HT-29 colon cancer cells.** HT-29 cells were plated at 3.8 × 10^6^ cells per T175 flask and grown to confluence. The cells were scraped into MBS containing either 0.5% Triton-X100, Brij58 or Brij97 on ice. The lysates were fractionated by sucrose density gradient centrifugation as described in Materials and Methods. Eleven 0.5 ml fractions were collected, with fraction 1 being the top and fraction 11 the bottom of the gradient. The fractions were analysed by Western blotting using the following antibodies (final concentrations in parentheses): CDCP1 (1 μg/ml goat ab1377), CD9 (2 μg/ml mouse monoclonal 602–29), TfR (100 ng/ml mouse monoclonal H68.4 (13–6800)), Flotillin-1 (50 ng/ml). **A, B:** Triton X-100 extraction; **C, D:** Brij58 extraction; **E, F:** Brij97 extraction. **B, D, F:** Quantitation of the Western blots shown in panels A,C and E respectively using Advanced Image Data Analyzer (AIDA) software.

DRM fractions were isolated using different detergents in order to characterise the nature of the membrane domain in which CDCP1 and CD9 reside. HT-29 cells were selected for DRM analysis as these cells have been extensively characterised in terms of lipid raft isolation and identification [29]. Classic lipid rafts were isolated using Triton-X100 detergent and sucrose density gradient centrifugation (Figure 3A, B). As expected, CD9 did not localise to Flotillin-1 positive (lipid raft) fractions [30, 31] and neither did CDCP1. In Triton-X100 extracts, CDCP1 and CD9 both localised to the denser, Transferrin Receptor (TfR)-containing non-raft associated fractions. When cells were lysed with Brij58, CD9 and CDCP1 were found in less dense fractions; the majority of CD9 (approx. 30-40%) being localised to fraction three and 10% of CDCP1 was also localised to this fraction (Figure 3C, D). Brij97 was more stringent, with only 6% of total CD9 protein localised to low density DRM fractions 1–4. Six per cent of total CDCP1 protein and four per cent of total TfR protein also localised to these fractions (Figure 3E, F). Interestingly, the peak fraction of CD9 immunoreactivity was located in fraction 8 while the peak of CDCP1 was located in fraction 9 (Figure 3E, F). Overall, this suggested that neither the CDCP1 nor the CD9 proteins were associated with lipid rafts, however, depending on the stringency of detergent lysis, co-fractionation of the two proteins in density gradients could be detected in cells lysed with Triton X100 or Brij58 detergents but this was much less pronounced when cells were lysed in Brij97.

### Protein complex formation between CDCP1 and CD9

**Figure 4:**
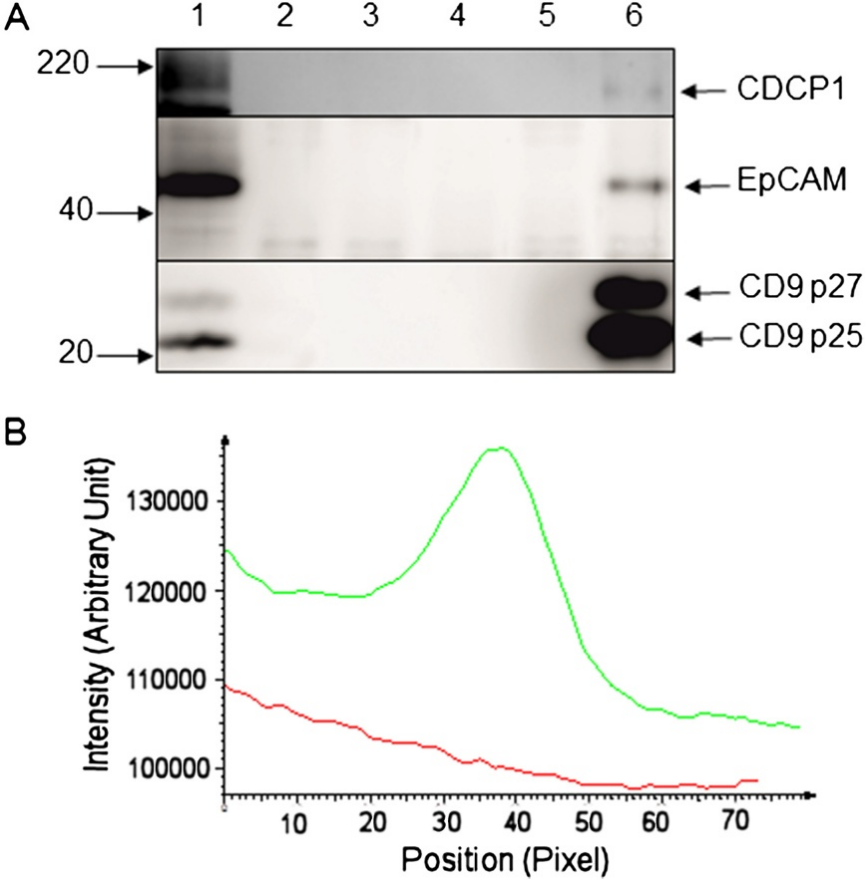
**Co-immunoprecipitation of CDCP1 and CD9.** SW480 cells were lysed in 1% (w/v) Brij97, 10 mM Tris–HCl (pH 7.4), 150 mM NaCl, 1 mM CaCl_2_, 1 mM MgCl_2_. **A.** Immunoprecipitation from whole cell extracts of SW480 cells was performed using 5 μg of ALB6 anti-CD9 monoclonal antibody (lane 6) or isotype-matched control antibody (lane 5). Immunoprecipitates were analysed by Western blotting with antibodies against either CDCP1 (ab1377), top panel; EpCAM (ab32392), middle panel or CD9 (602.29), lower panel. Further controls for the specificity of immunoprecipitation and Western blotting are: lane 1: 15 μg whole cell extract, lanes 2 and 3: 5 μg of isotype-control antibody and anti-CD9 antibody respectively; lane 4: SW480 proteins bound to protein G beads. **B.** Densitometric analysis of the Western blot for CDCP1. A portion of the blot corresponding to the CDCP1 p135 region was analysed in the tracks generated by immunoprecipitation with the control (red) and anti-CD9 (green) antibodies using AIDA software.

To explore a possible interaction between the CDCP1 and CD9 proteins, co-immunoprecipitation analysis was performed (Figure 4A). SW480 cells were selected as they expressed relatively high levels of both CDCP1 and CD9 proteins (Figures 1B and 2B). The cells were lysed in a buffer containing the Brij97 detergent which, despite partially separating CDCP1 and CD9 by density gradient centrifugation (Figure 3E, F), has previously been shown to preserve interactions between tetraspanins and partner proteins [21, 31]. The CD9 protein was immunoprecipitated with the ALB6 monoclonal antibody and protein G beads (Figure 4A, lower panel, lane 6). An immunoprecipitate formed using a control isotype-matched antibody (Figure 4A, lane 5) and protein G beads alone (Figure 4A, lane 4) were included as controls for non-specific immunoprecipitation. The control isotype antibody and the anti-CD9 antibody without either beads or lysate were also analysed by Western blotting to assess their contribution to the Western blot signals (Figure 4, lanes 2 and 3). The immunoprecipitates were subjected to Western blotting for CD9, CDCP1 and EpCAM (Epithelial cell adhesion molecule, a protein previously demonstrated to form a complex with CD9 [32]). As expected, EpCAM specifically co-immunoprecipitated with CD9 when the lysate was immunoprecipitated with CD9 antibody but not with an isotype-matched control antibody (Figure 4A, middle panel, lane 5). Importantly CDCP1 also co-immunoprecipitated with CD9 when the ALB6 CD9 antibody was used (Figure 4A, upper panel, lane 6) but not with the isotype-matched control (Figure 4A, upper panel, lane 5). The relevant portions of the lanes of the Western blot were analysed by densitometric scanning (Figure 4B). This showed a distinct peak of CDCP1 immunoreactivity in the track containing the anti-CD9 immunoprecipitate, however no peak was evident in the corresponding track formed using the control antibody.

## Discussion

The data described in this report has established a role for CDCP1 in colon cancer cell line adhesion and motility. A positive correlation between CDCP1 and CD9 protein expression in colon cancer cell lines was demonstrated. Reduction of CDCP1 expression in SW480 cells by RNA interference resulted in decreased cell motility, a finding consistent with previous reports on the effect of CDCP1 on cancer cell motility [3, 6]. In pancreatic and gastric cancer cells, CDCP1 phosphorylation and signalling appeared to be required for CDCP1-mediated migration [3]. Furthermore, in pancreatic cancer cells, binding of the δ isoform of protein kinase C (PKCδ) to CDCP1 tyrosine 762 facilitated cell migration [3]. PKCδ has been shown to promote cell migration in primary human keratinocytes, gastric cancer cell lines and, importantly, in colon cancer cell lines [33–35]. It will be important to investigate whether PKCδ signalling underlies CDCP1-mediated cell motility of SW480 cells.

The effect of CDCP1 on substratum adhesion of colon cancer cells was cell line-dependent. In Colo320 cells, ectopic expression of CDCP1 decreased cell binding to Matrigel, consistent with previous studies using engineered CDCP1 expression in HeLa cells [10]. This suggests that CDCP1 expression has negative effects on cell binding to ECM. In other studies, CDCP1 expression induced loss of cell adhesion to standard tissue culture plates in the MDA-468 breast cancer cell line [13]. However decreased Matrigel adhesion is not a universal feature that is associated with CDCP1 expression. For example, in the gastric cancer cell lines 44As3 and 58As9, reduced CDCP1 expression had no significant effect on cell binding to Matrigel but did increase cell binding to fibronectin [6]. Similarly, in our study, reduction of CDCP1 by RNA interference in SW480 cells had no significant effect on cell adhesion to Matrigel. Overall, these results indicate that the cell context may be critical for the effect of CDCP1 on cell-substratum adhesion.

Previous studies have suggested that CDCP1 and CD9 may interact within tetraspanin enriched microdomains (TEMs) [21]. Detergent resistance analysis performed in this study suggested that 6-10% of total CDCP1 protein was present within CD9-positive detergent resistant membrane fractions derived from HT-29 cells. An anti-CD9 antibody co-precipitated CDCP1 from SW480 cells lysed with Brij97 detergent, indicating that CDCP1 and CD9 may interact within Brij97-derived TEMs in HT-29 cells. This is in agreement with a study that used an anti-CD9 antibody to co-precipitate proteins from SW480 and SW620 cells and identified CDCP1 as a CD9-interacting protein using a proteomic approach [21]. The membrane co-fractionation and complex formation between CDCP1 and CD9 shown here suggests that tetraspanins could be important modulators of CDCP1-regulated functions.

## Conclusions

CDCP1 expression in colon cancer cell lines leads to modulation of cell:substratum adhesion and is required for maximal SW480 cell motility. Furthermore a molecular interaction between CDCP1 and CD9 was demonstrated which suggests that CD9 may modulate CDCP1 function.

## Electronic supplementary material

Additional file 1: Figure S1: Production of a CDCP1-expressing Colo320 cell line. **Figure S2.** Reduction of CDCP1 by RNA interference in SW480 cells. **Figure S3.** Reduction of SW480 colon cancer motility as a function of time after transfection of CDCP1 siRNA. (PDF 159 KB)

Additional file 2: Table S1: CDCP1 and CDP mRNA expression in a panel of colon cancer cell lines. CDCP1 and CD9 mRNA expression determined by microarray (as judged by Robust multi-array average (RMA)) from 47 colon cancer cell lines was downloaded from the Cancer Cell Line Encyclopedia [28]. (DOCX 20 KB)

## Abbreviations

CDCP1: CUB domain-containing protein 1
DMEM: Dulbecco’s modified eagle’s medium
DRM: Detergent-resistant membrane
ECM: Extracellular matrix
EpCAM: Epithelial cell adhesion molecule
FBS: Foetal bovine serum
HRP: Horseradish peroxidase
GAPDH: Glyceraldehyde-3-phosphate dehydrogenase
NGS: Normal goat serum
RTCA: Real-time cell analyser
siRNA: Small interfering RNA
TfR: Transferrin receptor
PKC: Protein kinase C.
