# Supplementary material for: CUB Domain Containing Protein 1 (CDCP1) modulates adhesion and motility in colon cancer cells
Source: BMC Cancer. 2014 Oct 9;14:754. doi: 10.1186/1471-2407-14-754 (PMC4200232; doi:10.1186/1471-2407-14-754)
Supplement: Supplementary file 1 — Additional file 1: Figure S1: Production of a CDCP1-expressing Colo320 cell line. Figure S2. Reduction of CDCP1 by RNA interference in SW480 cells. Figure S3. Reduction of SW480 colon cancer motility as a function of time after transfection of CDCP1 siRNA. (PDF 159 KB) [file 12885_2014_4938_MOESM1_ESM.pdf]

Introduction to Additional file 1 – further information relating to Figures S1-S3

Figure S1: Production of a CDCP1-expressing Colo320 cell line. Clones of G418-resistant Colo320 cells were produced as described in Methods. A. Clonal cell populations were screened for cell-surface CDCP1 expression by flow cytometry using the mouse monoclonal antibody CUB1. B. A selection of clones was further analysed by Western blotting using the anti-FLAG M2 antibody in Western blotting of whole cell extracts. Clone 12 was used for all further experiments since it displayed the highest level of cell-surface CDCP1 (assessed by flow cytometry) and total cellular CDCP1 (assessed by Western blotting).

Figure S2: Reduction of CDCP1 by RNA interference in SW480 cells. SW480 cells were plated at 2.2×10^5 cells per well in six well plates, cultured for approx. 15 h and transfected with 20pmoles of CDCP1 siRNA using Lipofectamine 2000. Three duplex siRNAs (at 8nM final concentration) were screened for reduction of CDCP1 (oligos 1–3). A) Cell-surface CDCP1 expression was assayed 48 hours post-transfection by flow cytometry using the CUB1 monoclonal antibody. B) To confirm the flow cytometry results shown in (A), total CDCP1 protein expression in SW480 cells at 48 hours post-transfection with 8nM siRNA (oligo 2) was analysed by Western blotting using the goat polyclonal anti-CDCP1 antibody. Oligo 2 (used in the remainder of this study) proved to be the most efficacious in reducing cell-surface and total cellular CDCP1.

Figure S3: Reduction of SW480 colon cancer motility as a function of time after transfection of CDCP1 siRNA. The motility of SW480 cells through an 8 μm pore membrane towards FBS following reduction of CDCP1 by RNA interference was measured using the Roche xCELLigence system. Data points at 1, 40, 60 and 72 h are shown. - = no addition; 10% serum =10% FBS was present in the lower chamber; Lipofectamine = Lipofectamine 2000 only; Control siRNA = Lipofectamine 2000 and control siRNA; CDCP1 siRNA = Lipofectamine 2000 and CDCP1 siRNA.

Supplementary Figure 1

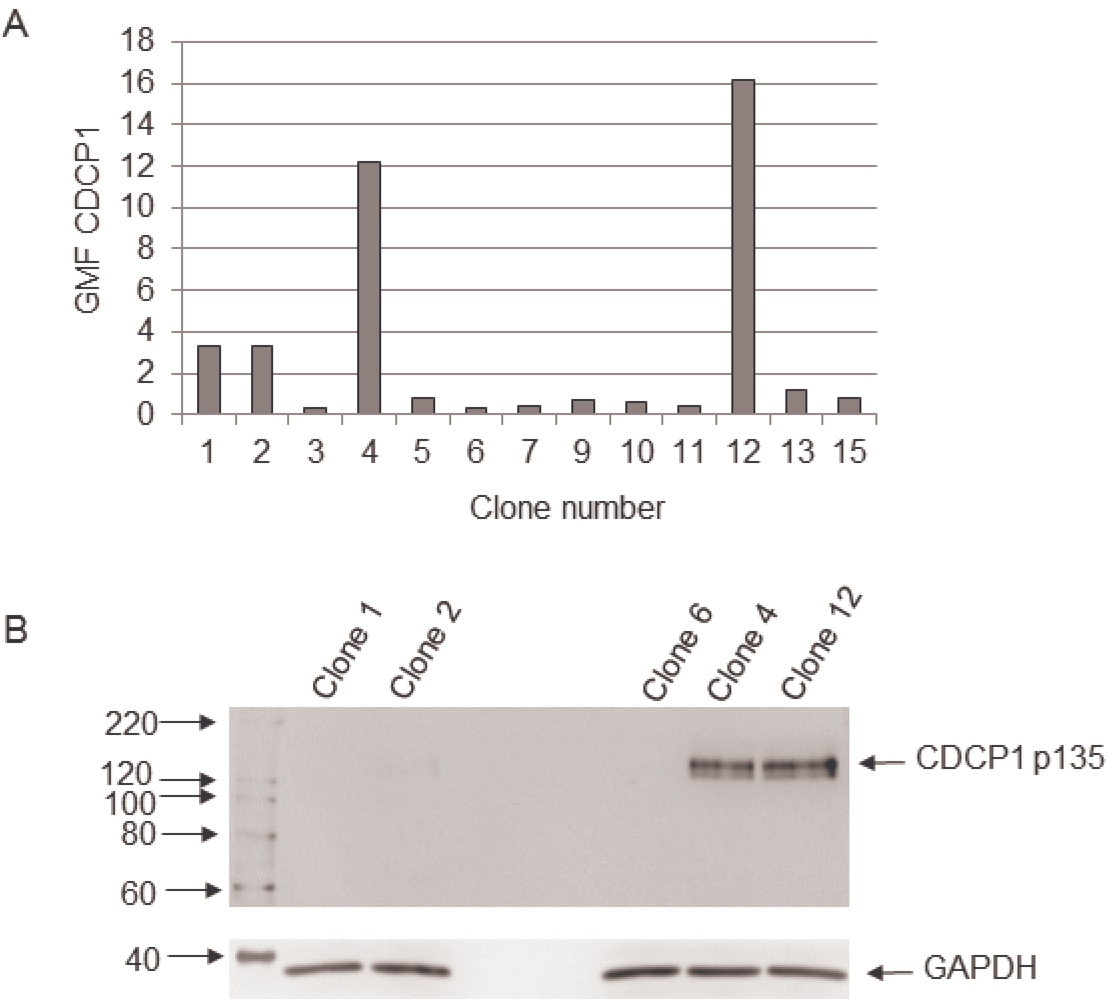

Supplementary Figure 2

A

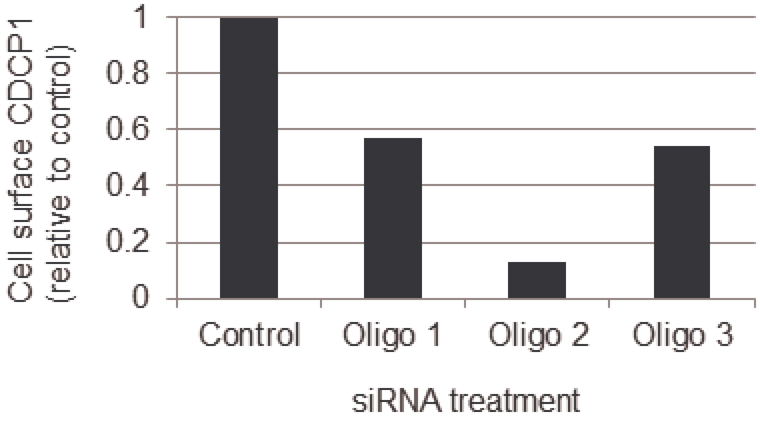

B

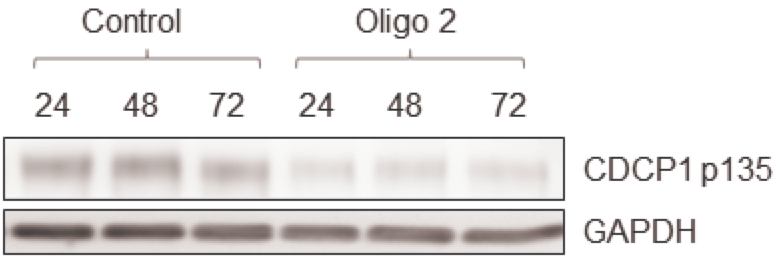

Supplementary Figure 3

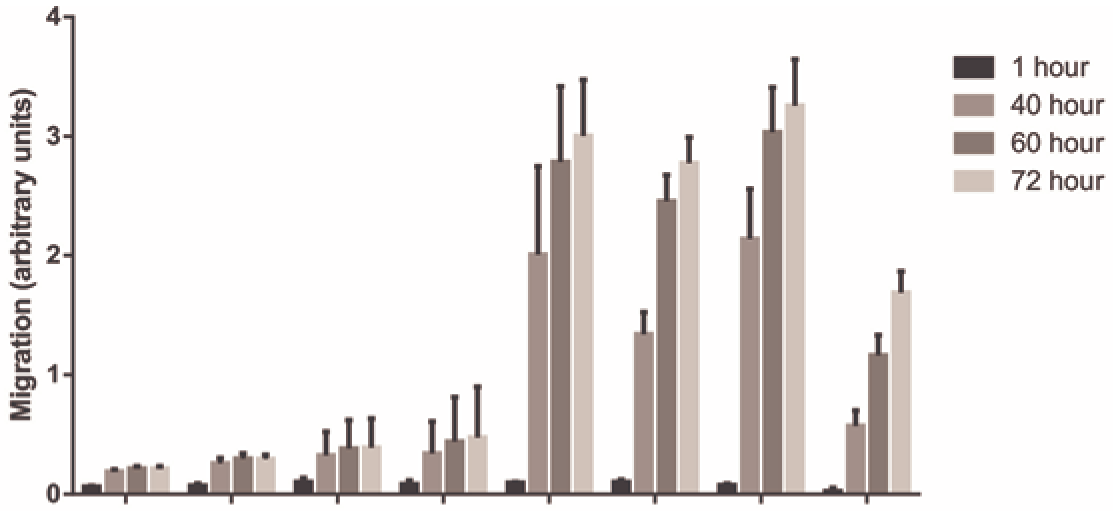

Chemo-attractant: 10% Serum	-	-	-	-	+	+	+	+
Lipofectamine	-	+	+	+	+	+	+	+
Control siRNA	-	-	+	-	-	-	+	-
CDCP1 siRNA	-	-	-	+	-	-	-	+
