# Supplementary material for: CUB Domain Containing Protein 1 (CDCP1) modulates adhesion and motility in colon cancer cells
Source: BMC Cancer. 2014 Oct 9;14:754. doi: 10.1186/1471-2407-14-754 (PMC4200232; doi:10.1186/1471-2407-14-754)
Supplement: Supplementary file 2 — Additional file 2: Table S1: CDCP1 and CDP mRNA expression in a panel of colon cancer cell lines. CDCP1 and CD9 mRNA expression determined by microarray (as judged by Robust multi-array average (RMA)) from 47 colon cancer cell lines was downloaded from the Cancer Cell Line Encyclopedia [28]. (DOCX 20 KB) [file 12885_2014_4938_MOESM2_ESM.docx]

**CDCP1 and CDP mRNA expression in a panel of colon cancer cell lines**

Supplementary Table 1

| **Cell line** | **Origin** | **CDCP1 RMA** | **CD9 RMA** |
| --- | --- | --- | --- |
| C2BBE1 | Large intestine | 6.82856 | 11.7852 |
| CCK81 | Large intestine | 5.99171 | 12.4741 |
| CL11 | Large intestine | 7.57557 | 12.7283 |
| CL34 | Large intestine | 6.71025 | 12.9879 |
| COLO205 | Large intestine | 6.36874 | 12.4563 |
| Colo320 | Sigmoid colon | 4.9164 | 4.14843 |
| COLO678 | Large intestine | 6.64812 | 13.707 |
| Colo741 | Pelvic wall metastasis | 4.85957 | 9.93511 |
| DLD1 | Large intestine | 7.19086 | 12.6139 |
| GP2D | Large intestine | 6.18772 | 13.122 |
| HCC56 | Large intestine | 6.78879 | 12.4436 |
| HCT116 | Large intestine | 8.36755 | 11.7225 |
| HCT15 | Large intestine | 6.90293 | 12.4482 |
| HS675T | Large intestine | 6.3079 | 12.1327 |
| HS698T | Large intestine | 4.80107 | 12.0538 |
| HT29 | Large intestine | 5.86422 | 11.6198 |
| HUTU80 | Small intestine | 4.95434 | 10.2711 |
| KM12 | Large intestine | 6.02328 | 11.5855 |
| LOVO | Large intestine | 6.0571 | 12.4157 |
| LS1034 | Large intestine | 6.7708 | 13.5833 |
| LS180 | Large intestine | 6.63448 | 12.7424 |
| LS411N | Large intestine | 6.23606 | 12.9321 |
| LS513 | Large intestine | 6.44925 | 13.3304 |
| NCIH508 | Large intestine | 6.71973 | 12.7621 |
| NCIH716 | Large intestine | 4.92405 | 8.25231 |
| NCIH747 | Large intestine | 6.81292 | 12.497 |
| OUMS23 | Large intestine | 6.25785 | 12.4879 |
| RCM1 | Large intestine | 8.02389 | 12.9107 |
| RKO | Large intestine | 6.82609 | 10.2828 |
| SKCO1 | Large intestine | 6.93385 | 13.3745 |
| SNU1040 | Large intestine | 6.21442 | 12.6283 |
| SNU175 | Large intestine | 6.45683 | 12.9142 |
| SNU283 | Large intestine | 6.75396 | 13.1733 |
| SNU407 | Large intestine | 6.14737 | 12.6174 |
| SNU503 | Large intestine | 6.4236 | 13.6116 |
| SNUC1 | Large intestine | 6.66072 | 12.0321 |
| SNUC2A | Large intestine | 6.10369 | 11.7764 |
| SW1116 | Large intestine | 6.76346 | 12.981 |
| SW1417 | Large intestine | 8.56398 | 13.1538 |
| SW1463 | Large intestine | 6.95447 | 12.4053 |
| SW403 | Large intestine | 5.89848 | 12.2616 |
| SW48 | Large intestine | 7.20712 | 13.0053 |
| SW480 | Large intestine | 7.40126 | 13.0027 |
| SW620 | Lymph node metastasis | 7.19693 | 12.5166 |
| SW837 | Large intestine | 5.95766 | 13.3902 |
| SW948 | Large intestine | 6.22248 | 12.905 |
| T84 | Large intestine | 7.10651 | 13.5129 |
